# Quercetin inhibits *Toxoplasma gondii* tachyzoite proliferation and acts synergically with azithromycin

**DOI:** 10.1186/s13071-023-05849-3

**Published:** 2023-08-03

**Authors:** Daniel A. Abugri, Sandani V. T. Wijerathne, Homa Nath Sharma, Joseph A. Ayariga, Audrey Napier, Boakai K. Robertson

**Affiliations:** 1 Department of Biological Sciences, College of Science, Technology, Engineering and Mathematics, Alabama State University, Montgomery, AL 36104 USA; 2 Microbiology PhD Program, Department of Biological Sciences, College of Science, Technology, Engineering and Mathematics, Alabama State University, Montgomery, AL 36104 USA; 3 Laboratory of Ethnomedicine, Parasitology and Drug Discovery, College of Science, Technology, Engineering and Mathematics, Alabama State University, Montgomery, AL 36104 USA

**Keywords:** Quercetin, Azithromycin, Combination, Synergizes, Inhibition, *Toxoplasma gondii*, Growth

## Abstract

Quercetin (QUE) is a natural polyphenol known to have numerous pharmacological properties against infectious and non-infectious diseases. Azithromycin (AZ) is an antibiotic that belongs to the azalide class of antimicrobials and an antiparasitic that is known to be effective in combination with clindamycin against pyrimethamine/sulfadiazine-resistant *Toxoplasma gondii* tachyzoites in clinical settings. Both compounds are known to target protein synthesis and have anti-inflammatory properties. However, little is known about QUE and AZ synergistic interaction against *T. gondii* growth. Here, we report for the first time the effects of the combination of QUE and AZ on *T. gondii* growth. The 50% inhibitory concentration (IC_50_) for QUE at 72 h of interaction was determined to be 0.50 µM, whereas AZ gave an IC_50_ value of 0.66 µM at 72 h of interaction with parasites. Combination testing of QUE and AZ in a ratio of 2:1 (QUE:AZ) showed an IC_50_ value of 0.081 µM. Interestingly, a fractional inhibitory index value of 0.28 was observed, indicating a strong synergy. QUE was also found to upregulate the generation of reactive oxygen species and cause dysfunction of the mitochondria membrane of both intracellular and extracellular *T. gondii* tachyzoites. Overall, the results indicate that QUE is a novel lead capable of synergizing with AZ for inhibiting *T. gondii* growth and may merit future investigation in vivo for possible combination drug development.

**Graphical Abstract:**

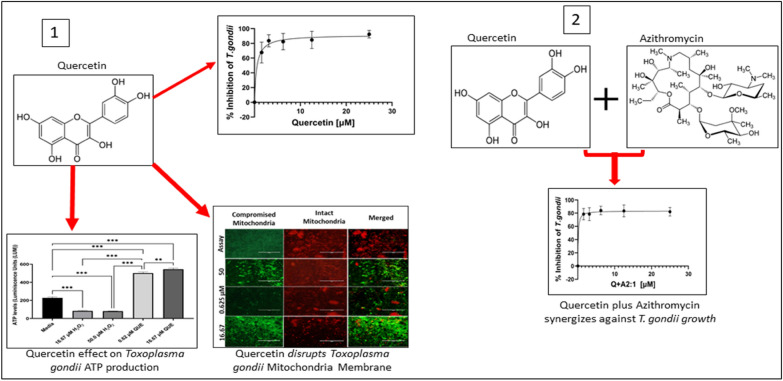

## Background

Toxoplasmosis is a global neglected parasitic disease caused by the obligatory intracellular protozoan parasite *Toxoplasma gondii*. The disease is known to affect both warm- and cold-blooded host cells [1–3] and it has been reported that more than one-third of the world population is infected with *T. gondii* [1, 3], including about 40 million people in the USA [4]. Also of major concern is that there are about 1.20 million cases of congenital toxoplasmosis across the world [5], with about 400 to 4000 cases reported annually in the USA [4]. The number may be higher since not all US states perform *T. gondii* seroprevalence testing in pregnant women. In fact, only a few states, such as Massachusetts and New Hampshire, screen for congenital toxoplasmosis during prenatal visits [6, 7].

*Toxoplasma gondii* infection in most immunocompetent individuals is clinically asymptomatic. However, immunocompromised people (e.g. HIV-infected patients, cancer patients, patients having organ transplants and those receiving blood transfusion) do present clinical signs that can range from mild to life threatening  [4, 8, 9].

*Toxoplasma gondii* is acquired through the ingestion of tissue cysts in raw or uncooked meat, contaminated soils, water and contaminated food products [2]. The two first-line treatment for *T. gondii* infection in humans are combination therapy with sulfadiazine (SDZ) and pyrimethamine (PYR) and combination therapy with trimethoprim and sulfamethoxazole [10, 11]. However, these drugs are associated with serious adverse health effects, such as induction of leucopenia, neutropenia, hypersensitivity reactions, thrombocytopenia, bone marrow suppression and megaloblastic anemia, in patients, with severely low platelet count [10–18]. These challenges necessitate more research into finding new anti-*T. gondii* inhibitors against toxoplasmosis that can work as sole therapy or in combination with other drugs.

Azithromycin (AZ) is a macrolide antibiotic that is a derivative of erythromycin and which has been extensively researched and proven to be an effective anti-*Toxoplasma* agent in the event of treatment failure with the pyrimethamine/sulfadiazine combination. This antibiotic is structurally similar to erythromycin, with high antibacterial properties and a desirable pharmacokinetic profile. AZ has been demonstrated to block translation in the normally translationally active plasmodial apicoplast and thus has been generally applied in treating pneumonia and chlamydia, especially in pregnant women [18]. Furthermore, AZ has been shown to control *T. gondii* infection in human villous explants [19].

Quercetin (QUE) is a polyphenolic flavonoid found in most plant foods, herbal supplements, vegetables, fruits, tea, red wines and seeds. It contains a broad spectrum of biological properties that have protective abilities, such as anti-inflammatory [20, 21], anti-mutagenicity [22], anti-cancer [23] and anti-oxidization [24] effects, inherent antibacterial properties against *Escherichia coli* [25] and reducing or preventing cardiovascular diseases [26]. Previous studies have shown that QUE synergizes with amoxicillin in the killing of *Staphylococcus epidermidis* [27], as well as with other antibiotics, such as with levofloxacin, ceftriaxone, gentamycin, tobramycin and amikacin against *Pseudomonas aeruginosa* [28] and with epigallocatechin gallate against *Leishmania* [29]. However, little is known about the interaction of QUE with AZ against *T. gondii* growth.

In the present study, we tested QUE alone and its combination with AZ, to test the hypothesis of whether synergy would result.

## Methods

### Culture of Vero cells and parasites

Vero cells (a lineage originally isolated from kidney epithelial cells extracted from an African green monkey) expressing firefly luciferase (Luc2p) were obtained from NIH Biodefense and Emerging Infections Research Resources Repository (BEI Resources, Manassas, VA, USA; NAID; NIH; NR-10385). * Toxoplasma gondii* type I virulent strain RH-(YFP)_2_, expressing yellow fluorescent protein (YFP)_2_, was kindly provided by Prof. William H. Witola (College of Veterinary Medicine, University of Illinois, Urbana Champaign, IL, USA). Vero cells were grown in a T-25-cm^3^ flask containing Dulbecco’s Modified Eagle Medium (DMEM) (Gibco, Thermo Fisher Scientific, Waltham, MA, USA) supplemented with 1% penicillin–streptomycin (PS) and amphotericin B solution as antibiotics (Gibco, Thermo Fisher Scientific) and 10% fetal bovine serum (FBS) (Life Technologies Inc., Thermo Fisher Scientific. Cultures were maintained at 37ºC with 5% CO_2_ to become confluent. *Toxoplasma gondii* tachyzoites of virulent strains RH-Wild-type (WT) with no fluorescent tag and hTERT (fibroblast) cells were provided by Prof. Silvia NJ Moreno (University of Georgia, Athens, GA, USA).

### Cytotoxicity of compounds

Vero cells (6 × 10^4^ cells/200 µl) were seeded into black 96 well plates and incubated for 24 h at 37 °C with 5% CO_2._ At 24 h, the Vero cells were washed to remove dead cells and 100 µl of growth media added to the cells. QUE and AZ were added in a 100-µl volume at concentrations of 25, 12.5, 6.25, 3.12, 1.56 and 0 µM, respectively, and incubated for 48 h. After 48 h, 10 µl Alamar blue dye (Abcam, Waltham, MA, USA) was added to the culture wells, and the plates were covered with aluminum foil and incubated at standard culture conditions. The fluorescence was measured at 560/630 nm using the Tecan 200F infinite fluorescent plate reader (Tecan Group, Männedorf Switzerland). Experiments were performed in three independent experiments and the results presented as the mean ± standard deviation (SD) (*n* = 3).

### In vitro* T. gondii* growth

The QUE and AZ were obtained from Santa Cruz Biotechnology Inc. (Dallas, TX, USA and prepared in dimethyl sulfoxide (DMSO). The *T. gondii* RH-(YFP)_2_ tachyzoites expressing yellow fluorescent protein (YFP)_2_ throughout culture were used to test the inhibitory effect of QUE alone and in combination with AZ on *T. gondii* parasites in vitro. Vero cells (6.0 × 10^4^ cells/(200 µl) were seeded into 96-well plates and incubated at 37 °C with 5% CO_2_ for 24 h for 90% confluence, following which precisely 100 µl of freshly purified tachyzoites at a concentration of 1 × 10^4^ parasites/well was added to the Vero cells. The experimental compound QUE and the standard drug (AZ) were added in a volume of 100 µl at concentrations 0, 1.56, 3.12, 6.25, 12.5 and 25 µM, respectively, and incubated at 37 °C with 5% CO_2_. *Toxoplasma gondii* growth at 72 of culture was measured using Tecan 200 F infinite fluorescent plate reader with excitation set at 485 nm and emission set at 535 nm (Tecan Group). The fluorescent intensities were converted into percentage inhibition using the formula reported in [30]. The concentrations of the compound QUE and AZ were plotted against the percentage inhibition of *T. gondii* growth using GraphPad Prism software 9.2 (GraphPad Software, San Diego, CA, USA. Experiments were conducted in three independent experiments and the results presented as the mean ± SD (*n* = 3).

### Reversibility of *T gondii* after drug withdrawal

At 72 h post-treatment with QUE and AZ, the plates were washed 3 times with 1× phosphate-buffered saline (PBS) to remove all compounds and all extracellular parasites. After washing, 100 µl of 10% FBS-supplemented DMEM was added to each well, followed by initial reading as day 0. Parasite fluorescence intensity was recorded for the following 72 h, and the 50% effective minimum concentrations were determined at 72 h to determine whether the compounds removed still had any effect on tachyzoites growth. The negative control wells (where no drugs were previously added) served as the 100% benchmark and the post-treatment with QUE and AZ withdrawal served as the experimental wells. The IC_50s_ values were determined as stated above in the individual growth inhibition assay. Experiments were conducted in three independent experiments and the results presented as the mean ± SD of triplicate trials (*n* = 3).

### Combination chemotherapy with QUE and AZ

Vero cells (6.0 × 104 cells/200 µl) were seeded into 96-well plates for 24 h, as described above for the testing of individual compounds, followed by purification of RH-(YFP)_2_ tachyzoites as described above. The concentration of parasites used in the individual studies described above was added at a volume of 100 µl, followed by the addition of drugs in a ratio of 1:1 (50 µM QUE: 50 µM AZ), 2:1 (50 µM QUE: 25 µM AZ) and 1:2 (25 µM QUE: 50 µM AZ), serially diluted, and parasite growth was monitored using the Tecan 200F infinite fluorescent plate reader (Tecan Group) with the filters set to 458 nm/535 nm as excitation and emission wavelengths, respectively. Parasite growth was monitored for 72 h, and the IC_50s_ values were calculated from the interaction data at 72 h using GraphPad prism software version 9.2.0 (GraphPad Software). Experiments were conducted in three independent experiments and the results presented as the mean ± SD of triplicate trials (*n* = 3). To decipher the combination that could exert synergy and addictiveness for future modification, we used the fractional inhibitory concentration (FICI). Synergy was defined as an inhibition IC_50_ produced by a combination of compounds (QUE-AZ) that is greater than the sum of the inhibitory concentration effects produced by QUE or AZ alone. FICI values were determined the using formula reported in [31–33] : FICI = IC_50p_ of QUE in combination (QUE-AZ [1:1, 1:2 or 2:1]/IC_50p_ of QUE alone + IC_50p_ of AZ in combination (QUE-AZ [1:1, 1:2 and 2:1])/IC_50p_ of AZ alone. We used a standard guideline which states that FICI values < 0.5 are considered to be synergistic; FICI values ≥ 1 indicate an additive and FICI values ≥ 2 indicate an antagonistic interaction [31].

### Measurement of reactive oxygen species in* T. gondii*

QUE has been previously reported to induce reactive oxygen species (ROS) production in *Leishmania amazonensis*. To verify whether QUE had any effect on ROS production in *T. gondii* tachyzoites, we used a procedure similar to that reported in an earlier study [30] with modifications. WT *T. gondii* tachyzoites provided by Silvia NJ Moreno (University of Georgia, Athens, GA, USA) were maintained in growth media without phenol red in intact cells. We harvested freshly lysed RH-Wild type (RH-W) parasites by passing them through a 27-gauge needle followed by filtration through a 3-µm filter. RH-W parasites (1.60 × 10^6^ parasites/50 µl per well) were seeded into black 96-well plates and treated either with H_2_O_2_ (500 µM) as a positive control [34] or with QUE at different concentrations (0.66 and 12.5 µM), for 30 min at 37 °C with 5% CO_2_. After a 30-min incubation, ROS dye (Abcam) was added and the wells incubated for a further 45 min according to the manufacturer’s protocol and previous work [35]. Fluorescence intensities of the wells were measured at an excitation of 485 nm and emission at 563 nm, respectively, using the Tecan 200F infinite microplate reader (Tecan Group). Experiments were conducted in three independent experiments and the results presented as the mean ± SD of triplicate trials (*n* = 3).

### QUE disrupts *T. gondii* mitochondrial membrane potential

For mitochondrial membrane potential (ΔΨm) measurements, we used the cationic JC-1 dye as a fluorescent probe (Abcam), as previously described [30, 36]. This membrane potential kit has been widely used in *T. gondii* tachyzoite mitochondrial membrane potential testing in vitro [35, 37–40], with modifications. We measured both the intracellular and extracellular ΔΨm of the parasite. For the intracellular assay, 6 × 10^4^ Vero cells were seeded into wells of a black 96-well plate with a clear bottom (Costar, Corning Inc., Corning, NY, USA) until they were confluent. Then 6 × 10^4^ tachyzoites from *T. gondii* RH-WT strains were added. In contrast, in the assay for extracellular parasites, 1 × 10^5 ^freshly purified parasites in 100 μl media were seeded directly into the wells (no host cells). Then, either 100 μl solution containing 0.625, and 16.67 μM of QUE as the experimental drug, 50 μM of carbonyl cyanide* m*-chlorophenyl hydrazone (CCCP; Alfa Aesar, Haverhill, MA, USA) as a positive control or 1× HBSS (assay buffer) the negative control was added to the designated wells and incubated for 8 h at 37 °C with 5% CO_2 _ [30, 36]. A 10-μl aliquot of JC-1 was added to the wells, and the plates were covered with aluminum foil and incubated for 45 min. The solutions were then removed and centrifuged at 12 °C, 2000 rpm for 5 min, and the supernatant was removed. Next, 100 μl of assay buffer was added to each well and centrifuged again under the same conditions and the supernatant discarded. The parasite pellets were resuspended in solution with 100 μl of assay buffer for the extracellular assay. For the intracellular assay, the supernatant was discarded followed by the addition of 100 μl of assay buffer. Both intracellular and extracellular parasite assays were imaged using an EVOS FL fluorescence microscope (Invitrogen Life Technologies, Thermo Fisher Scientific). The experiments were performed in triplicate (*n* = 3) [36].

### QUE effect on *T. gondii* adenosine triphosphate production

To further validate the results of our mitochondria assay, we used a modified protocol [30, 37, 39]. Fresh extracellular RH-RFP parasites (2.84 × 10^4^) were incubated in a complete medium containing QUE at concentrations of 0.62 and 16.67 µM, in complete medium without the drug (medium only with parasites as negative control) or in medium containing 500 µM H_2_O_2_ as a negative control. After 8 h of incubation under the standard culture conditions of 5% CO_2_ at 37 °C, the ATP Detection Assay Kit - Luminescence (catalog no. 700410; Cayman Chemical, Ann Arbor, MI, USA) was used to measure the luminescence of samples. QUE (the drug treated), the positive control (H_2_O_2_) and the negative control (medium with parasites without drugs) were washed twice with PBS and the pellets lysed on ice with 100 µl of lysis buffer. Next, 10 µl of the parasite’s lysates were added to 100 µl of adenosine triphosphate (ATP) detection working solution in each opaque microplate’s well (Corning). The plates were incubated for 20 min at room temperature according to the protocol of the ATP Detection Assay Kit, the covers of the plates were removed and luminescence was measured using the BioTek Cytation cell imaging multi-mode microplate reader with software Gen 5.3.1 (Agilent Technologies Inc., Santa Clara, CA, USA). The effect of treatments on *T. gondii* ATP luminescence was compared with ATP luminescence of untreated *T. gondii* population (medium with parasites without experimental drugs or negative controls) and expressed as percentages of these control ATP values. Experiments were conducted in three independent experiments and the results presented as the mean ± SD of triplicate trials.

### Statistical analysis

GraphPad Prism software (GraphPad Software) was used to determine the IC_50_ values. A one-way analysis of variance (ANOVA) analysis was used to distinguish any statistical differences. A *p* value of 0.05 was considered to indicate significance.

## Results and discussion

### In vitro inhibition of *T. gondii* growth

The IC_50_ values for QUE and AZ against *T. gondii* tachyzoite growth at 72 h were determined to be 0.50 µM and 0.66 µM, respectively (Table 1).

**Table 1:** Comparison of the in vitro inhibitory activity of azithromycin and quercetin against *Toxoplasma gondii* growth

Compounds (µM)	IC_50_	CC_50 Vero cells_	SI_Vero cells_	FIC	Designation
QUE Alone	0.50	14.98	29.96		
AZ Alone	0.66	2575.00	3901.5		
QUE + AZ (2:1)	0.08	60.82	760.25	0.28	Synergy
QUE + AZ (1:2)	1.50	47.17	31.45	5.27	Antagonistic
QUE + AZ (1:1)	0.89	37.22	41.82	3.13	Antagonistic

Data in table are the means IC_50s_ of *Toxoplasma gondii* strain RH-(YFP)_2_ tachyzoites from at least 3 experiments

*IC*_*50*_ 50% inhibitory concentration,* CC*_*50*_ 50% cytotoxicity concentrations,* SI *selectivity index (CC_50 Vero cells_/IC_50_* T. gondii*),* FIC* fractional inhibitory concentration: IC_50_ of QUE in combination (QUE-AZ (1:1, 1:2 and 2:1)/IC_50_ QUE alone + IC_50_ of AZ in combination (QUE-AZ (1:1) or (1:2) or (2:1)/IC_50_ of AZ alone,* QUE* quercetin,* AZ* azithromycin

**Fig. 1:**
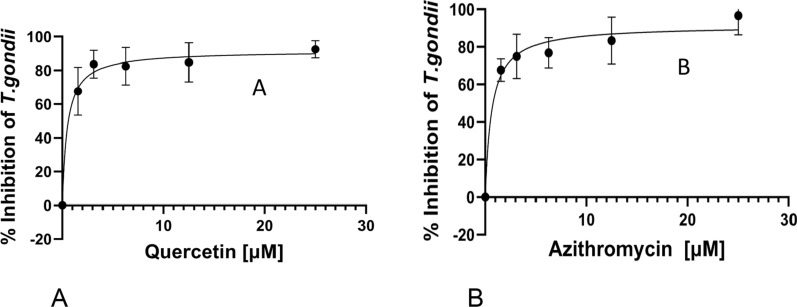
*Toxoplasma gondii* growth inhibition curves at 72 h in the presence of quercetin (**a**) and azithromycin (**b**) at concentrations of 0, 1.56, 3.12, 6.25, 12.5 and 25 µM, interaction. Data are presented as means (filled circles) of three independent experiments performed in triplicate, with the standard error of the mean (whiskers)

The growth curves of the parasite, as percentage growth inhibition, in the presence of different concentrations of AZ and QUE, respectively, are presented in Fig. 1a, b. Interestingly, the IC_50s_ values in our current study for both individual compounds are comparatively similar to those typical of the currently used drugs PYR and SDZ reported using HFF cells (0.95–1.55 µM for SDZ and 2.42–3.52 µM for PRY [30]; 1.17 ± 0.076 for PRY [41]); 0.79–1.5 µM for PYR [42]; 3.4 µM for PYR [32]; 0.8 µM for PYR [43]; 0.16 µM for PYR [44]).

### QUE interaction with AZ in vitro

**Fig. 2:**
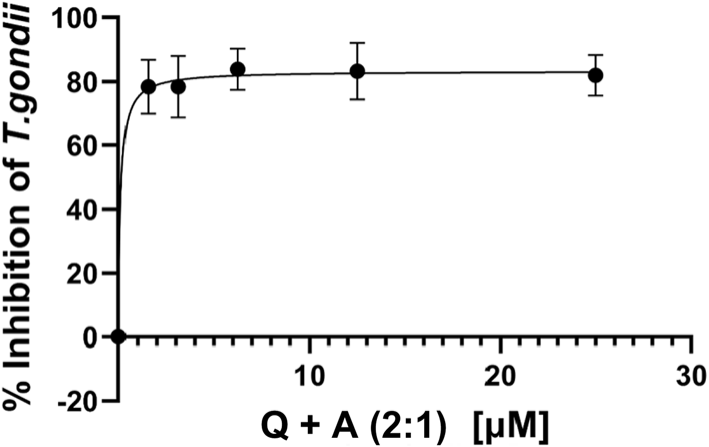
In vitro *T. gondii* strain RH-(YFP)_2_ inhibitory growth curve at 72 h in the presence of combined quercetin (QUE) and azithromycin (AZ) at the ratio of 2:1 (QUE: AZ) (50 µM QUE: 25 µM AZ). Concentrations tested were 50, 25, 12.5, 6.25, 3.125 and 0 µM for QUE, and 25, 12.5, 6.25, 3.125, 1.56, and  µM for AZ. Data are presented as the means (filled circles) of three independent experiments performed in triplicate, with the standard error of the mean (whiskers). Q+A (2.1), Quercetin: azithromycin at the ratio of 2:1

Combined treatment with QUE and AZ was performed to determine whether these drugs could synergize against *T. gondii* growth in vitro. We noted that the combined treatment with QUE and ZA at a ratio of 2:1 (QUE: AZ) showed a strong synergy at 72 h after treatment initiation. The IC_50s_ values for combined treatment with QUE and ZA at a ratio of 2:1 (QUE: AZ), 1:2 (QUE: AZ) and 1:1 of (QUE: AZ) were determined to be 0. 89, 1.50 and 0.081 µM, respectively (Table 1). The fractional inhibitory concentrations were calculated to be 0.28. The ratio of 2:1 (QUE: AZ) was strongly synergistic based on the FICI calculated (Table 1). However, combined treatment with QUE and AZ at ratios of 1:2 (QUE: AZ) and 1:1 (QUE: AZ) resulted in antagonistic interactions. The growth curve for the 2:1 ratio of QUE: AZ is shown in Fig. 2.

### Cytotoxicity and selectivity index

The 50% cytotoxic concentrations (CC_50_) were calculated to be 14.98 µM for QUE, 2575 µM for AZ, 60.82 µM for QUE+AZ(2:1), 47.17 µM for QUE+AZ (1:2) and 37.22 µM for QUE+AZ (1:1) (Table 1). The selectivity indices (SI) for QUE, AZ and QUE+AZ (2:1) were calculated and are presented in Table 1. The SI values for the 1:1, 1:2 and 2:1 ratios of the QUE+AZ combinations were calculated to be 31, 42, and 760.25, respectively. All of the individual compounds and ratios tested had a broad-spectrum inhibitory effect on parasite growth in vitro compared to some of the current drugs used in the clinical setting to treat *T. gondii* infection.

### Individual compounds withdrawal effect on parasite growth

**Fig. 3:**
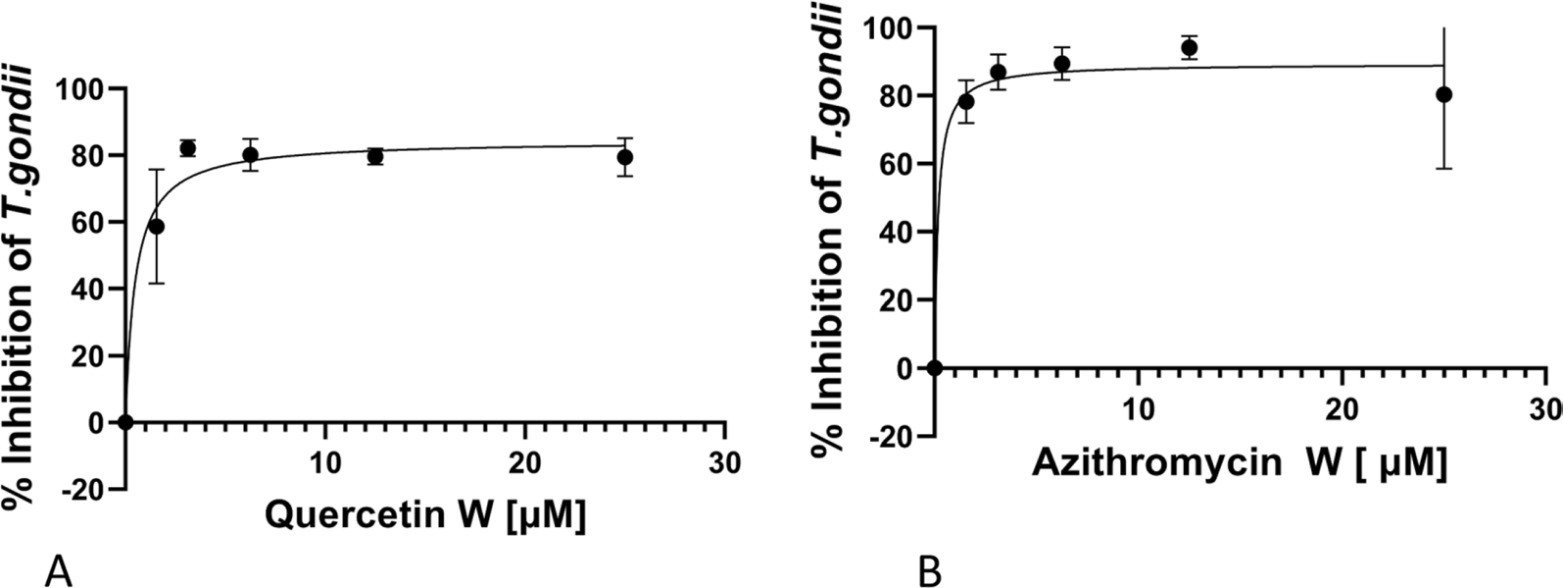
*Toxoplasma gondii* inhibition curves for parasites first treated for 72 h and then grown in growth media for 72 h after drug withdrawal. **a** and **b** Depict growth curve of Quercetin and Azithromycin withdrawal treatment. Data are presented as the mean (filled circles) of three independent experiments performed in triplicate, with the standard error of the mean (whiskers)

To decipher whether the withdrawal of QUE after 72 h of treatment had any effect on the continued growth of tachyzoites during the first 72 h post-drug withdrawal, we determined the curves for parasite inhibition (Fig. 3a, b). It was noted that withdrawal of the compound did not abolish its inhibitory effect on parasite growth relative to the standard drug (AZ) tested at 72 h (Table 2). The IC_50s_ values were calculated to be 0.49 and 0.20 µM for QUE and AZ at 72 h, respectively (Table 2).

**Table 2:** Comparison of inhibitory concentration of RH-(YFP)_2_ *Toxoplasma gondii* tachyzoites after drug withdrawal at 72 h

Compounds (µM)	72 h
Que	0.49 ± 0.10
AZ	0.20 ± 0.11

Data are presented as the means ± standard deviation of RH-(YFP)_2_ tachyzoites from at least triplicate experiments performed in triplicates

AZ has also been known to have an effect on a secondary target of parasites through the apicoplast [45, 46]. Specifically, it has been reported that AZ affects lipid levels and membrane lipid fluidity in cells [47, 48]. Taken together, our findings and previous data suggest that AZ could affect sphingolipid and phospholipid production in the apicoplast, which is crucial for the parasite lytic cycle.

QUE causes high ROS production and mitochondrial membrane disruption, which may affect lipid synthesis (e.g. phospholipids and sphingolipids) directly or indirectly in the apicoplast and the mitochondria. Lipids are crucial for *T. gondii* invasion, proliferation and modulation of intracellular calcium that triggers parasites egress and controls most of the lytic cycle activities in the *T. gondii* parasite. However, this conjecture requires further study.

Our observation of the effect of AZ remaining active against tachyzoite growth even after drug withdrawal confirmed its long-acting ability due to its high half-life > 50 h in host cells [49].

### Mitochondria membrane potential, ATP production and ROS production in intracellular parasites

**Fig. 4:**
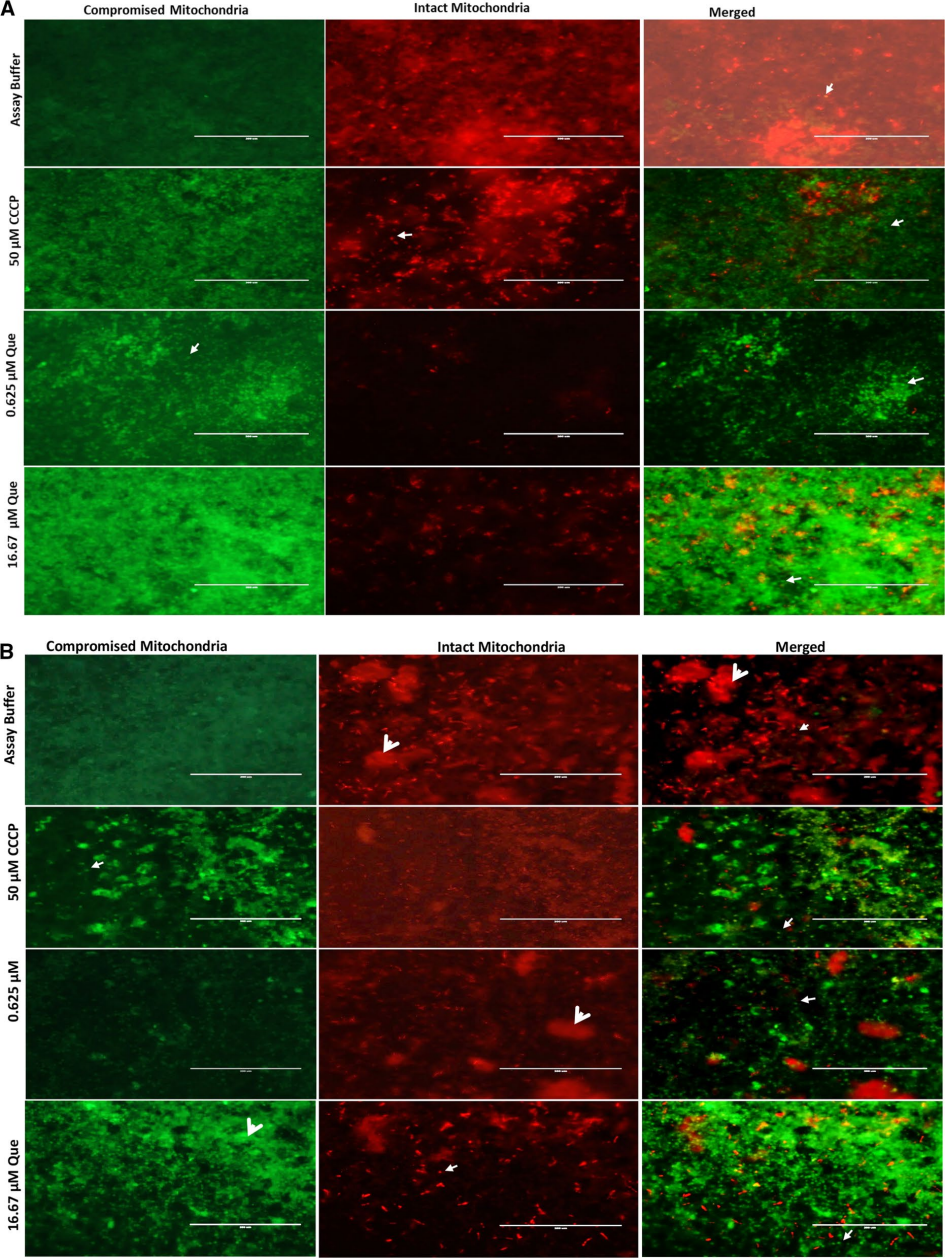
**a** Quercetin disrupts the mitochondria membrane potential (MMP) in extracellular *T. gondii* tachyzoites. White arrows indicate parasites. The panel with red represents the uncompromised MMP in parasites and the panel with green indicates the compromised MMP in parasites treated with Que (0.62 and 16.67 µM) and a standard compound (CCCP) at 50 µM. The green indicates compromised MMP. Scale bar: 200 µm. **b** Que disrupts the MMP in intracellular *T. gondii* tachyzoites. Small white arrows indicate parasites, and short large arrowheads indicate host cells. The red spikes indicate intact mitochondria in tachyzoites treated with Que (0.62 and 16.67 µM) and a standard compound CCCP at 50 µM. The green indicates the compromised MMP. Scale bar: 200 µm. CCCP, Carbonyl cyanide m-chlorophenyl hydrazone; Que, quercetin

**Fig. 5:**
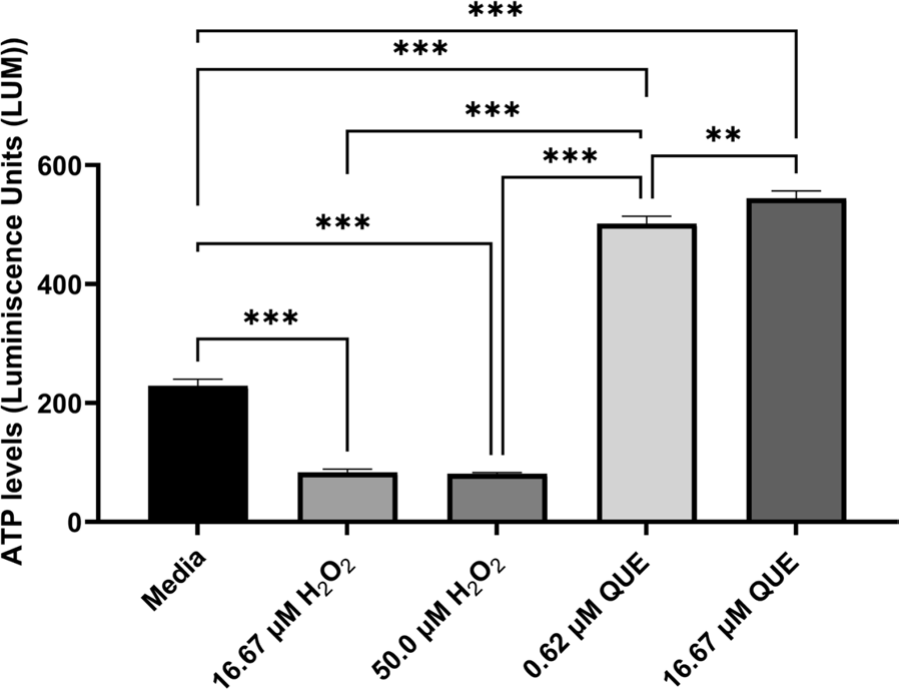
Quercetin disrupts ATP production in extracellular *T. gondii* tachyzoites. QUE, Quercetin. **  and *** indicates a statistical difference between treatments at *p* < 0.01 and *p* < 0.001 respectively

**Fig. 6:**
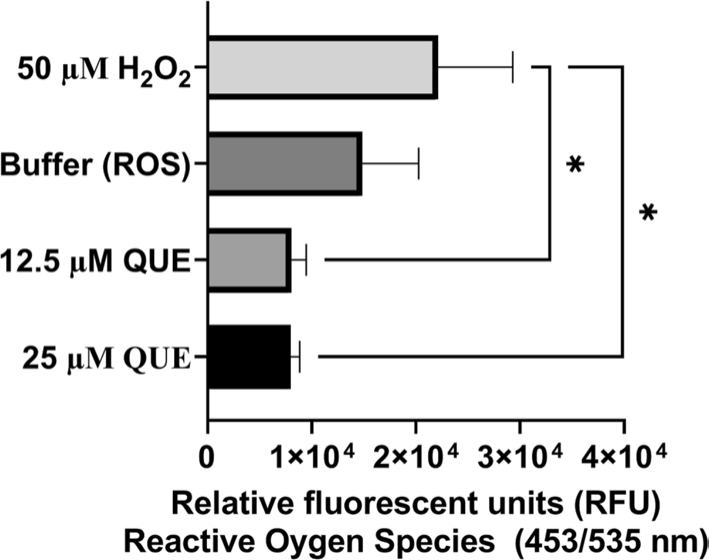
Quercetin induces ROS production in intracellular *T. gondii* tachyzoites. H_2_O_2_, Hydrogen peroxide; QUE, quercetin; ROS, reactive oxygen species. * indicates *p* < 0.05

 The mitochondria is considered to be the powerhouse of eukaryotic cells, performing various functions, including ROS mediation, ATP production, fatty acid synthesis, translation and transcription [50]. To better understand the mechanism of action of QUE on ΔΨm, ATP production and ROS production, we employed assays that measured intracellular parasite responses to QUE in a dose-dependent manner. The results showed the effect of QUE on ΔΨm (see Fig. 4a). Similarly, we observed that QUE disrupted extracellular parasite ΔΨm (Fig. 4b). Also, to explore the effect of QUE on parasite ATP production in vitro, we performed an ATP production assay (see results of assay in Fig. 5). Study of the generation of ROS was also carried out to determine the effect of QUE on ROS released (see results of assay in Fig. 6). We observed slight statistical differences between 25 µM of QUE versus 50 µM of H_2_O_2_ (*p* < 0.026) and 12.5 µM of QUE versus 50 µM of H_2_O_2_ (*p* < 0.026), confirming results reported in a previous study in *Leishmania amazonensis* which showed that QUE causes ROS production and mitochondrial dysfunction [50]. Also, several studies have shown that compounds that lead to ΔΨm disruption and high ROS production could lead to disruption of ATP production in parasites [30, 37, 39, 51]. Furthermore, compounds exerting high ROS production and mitochondria membrane depolarization lead to peroxidation of long-chain fatty acids and the production of toxic intermediates, such hexanal, aldehyde and alkenes in host cells and parasite cells, further resulting in apoptotic activities and eventually cell death [52, 53]. We observed a statistical difference between the treatments with QUE, H_2_O_2_ (positive control) and medium alone (negative control). Confirming the results of previous studies reported in *Lesihmania* spp, using QUE, our current study showed a statistical difference between the standard positive control (50 µM H_2_O_2_) versus 0.62 µM QUE (*p* < 0.001), 16.67 µM QUE versus 0.62 µM QUE (*p* < 0.001), medium versus 50 µM H_2_O_2_ and 16.67 µM QUE versus 0.62 µM QUE (*p* < 0.001). Interestingly, the IC_50_ of QUE alone and in combinations with AZ were observed to be effective at submicrolar concentrations, which is in contrast to the results reported for *Leishmania* spp. [50]. This difference could be attributed to the host cell types used as a medium of propagation of the parasites, the concentration of QUE and/or the number of parasites used. It has also been reported that QUE is highly anti-inflammatory and antioxidant, and these properties have been associated with its oxidative, kinase and cell-cycle inhibition [54]. Studies have also shown its ability to induce apoptosis in cancer cells [55]. Our previous work using dihydroquercetin showed *T. gondii* inhibition but not as effective as QUE [32], possibly partly due to the number of hydroxyl groups available in QUE and the analog dihydroquercetin.

## Conclusion

In conclusion, we found that QUE inhibited tachyzoite growth and also caused disruption of the mitochondrial membrane potential, depletion of ATP and increased ROS production. Any organelle disruption could directly or indirectly affect energetics and many other pathways, depending on the mitochondria-directed pathways. Comparatively, the combination therapy (AZ + QUE) was more effective against parasite proliferation at a ratio of 2:1 (QUE: AZ) than each of the individual compounds alone. This implies that QUE might be a good candidate for future combination with AZ. In vivo testing to ascertain its efficacy and safety will be an exciting and necessary investigation to undertake.

## Data Availability

All data has been included in the manuscript and raw data are available upon request.

## Abbreviations

AZ: Azithromycin
IC_50_: Half-maximal inhibitory concentration
QUE: Quercetin
